# A high-performance approach for predicting donor splice sites based on short window size and imbalanced large samples

**DOI:** 10.1186/s13062-019-0236-y

**Published:** 2019-04-11

**Authors:** Ying Zeng, Hongjie Yuan, Zheming Yuan, Yuan Chen

**Affiliations:** 1 Hunan Engineering & Technology Research Center for Agricultural Big Data Analysis & Decision-making, Hunan Agricultural University, Changsha, 410128 Hunan China; 2 Orient Science & Technology College, Hunan Agricultural University, Changsha, 410128 Hunan China; 3 Hunan Provincial Key Laboratory for Biology and Control of Plant Diseases and Insect Pests, Hunan Agricultural University, Changsha, 410128 Hunan China; 4 Hunan Provincial Key Laboratory of Crop Germplasm Innovation and Utilization, Hunan Agricultural University, Changsha, 410128 Hunan China

**Keywords:** Donor splice site, Short window size, χ^2^-DT, Chi-square test, Balanced decision table

## Abstract

**Background:**

Splice sites prediction has been a long-standing problem in bioinformatics. Although many computational approaches developed for splice site prediction have achieved satisfactory accuracy, further improvement in predictive accuracy is significant, for it is contributing to predict gene structure more accurately. Determining a proper window size before prediction is necessary. Overly long window size may introduce some irrelevant features, which would reduce predictive accuracy, while the use of short window size with maximum information may performs better in terms of predictive accuracy and time cost. Furthermore, the number of false splice sites following the GT–AG rule far exceeds that of true splice sites, accurate and rapid prediction of splice sites using imbalanced large samples has always been a challenge. Therefore, based on the short window size and imbalanced large samples, we developed a new computational method named chi-square decision table (χ^2^-DT) for donor splice site prediction.

**Results:**

Using a short window size of 11 bp, χ^2^-DT extracts the improved positional features and compositional features based on chi-square test, then introduces features one by one based on information gain, and constructs a balanced decision table aimed at implementing imbalanced pattern classification. With a 2000:271,132 (true sites:false sites) training set, χ^2^-DT achieves the highest independent test accuracy (93.34%) when compared with three classifiers (random forest, artificial neural network, and relaxed variable kernel density estimator) and takes a short computation time (89 s). χ^2^-DT also exhibits good independent test accuracy (92.40%), when validated with BG-570 mutated sequences with frameshift errors (nucleotide insertions and deletions). Moreover, χ^2^-DT is compared with the long-window size-based methods and the short-window size-based methods, and is found to perform better than all of them in terms of predictive accuracy.

**Conclusions:**

Based on short window size and imbalanced large samples, the proposed method not only achieves higher predictive accuracy than some existing methods, but also has high computational speed and good robustness against nucleotide insertions and deletions.

**Reviewers:**

This article was reviewed by Ryan McGinty, Ph.D. and Dirk Walther.

**Electronic supplementary material:**

The online version of this article (10.1186/s13062-019-0236-y) contains supplementary material, which is available to authorized users.

## Background

The amount of genomic sequence data has increased exponentially as a result of the advancement in sequencing technology. Therefore, there is an urgent need to complete genome annotation quickly and reliably. Gene identification is an important task in genome annotation. Most eukaryotic genes consist of protein-coding regions (exons) and noncoding regions (introns), with the exons being separated by intervening introns [1]. The boundaries between exons and introns are called splice sites and are the locations where RNA splicing occurs. The 5′ end of an intron is a donor splice site and the 3′ end is an acceptor splice site. If we can accurately detect splice sites, the coding regions of DNA sequences can be located, so splice site prediction plays a key role in gene identification. Almost 99% of splice sites are canonical GT–AG pairs [2], that is, dinucleotides GT and AG for donor and acceptor splice sites, respectively. However, this strong conservation observed in splice sites is not sufficient to accurately identify them, due to the abundance of dinucleotides GT and AG appearing at non-splice site positions. We therefore face an extremely imbalanced classification task, namely, the discrimination of small numbers of true splice sites from much larger volumes of decoy positions with the dinucleotides GT and AG [3].

For splice site prediction based on machine learning approaches, the main steps are feature extraction and classifier selection or design. The extracted features are usually based on nucleotide position information [4–9], the frequency of *k*-mers [4, 6, 10], dependence between adjacent and nonadjacent nucleotides [1, 6, 11–13], RNA secondary structure information [14–18], DNA structural properties [19], and some other attributes that can be calculated directly from sequence information [20–22]. The commonly used classifiers include support vector machine (SVM) [1, 3, 5, 6, 10, 18, 23–25], artificial neural network (ANN) [26–29], random forest (RF) [13], and decision tree [30].

Although relatively high accuracy has been achieved with the methods currently available (e.g., the accuracy for most donor splice site prediction based on the HS^3^D dataset has exceeded 90% [6, 10, 12, 13, 19, 24, 31]), further study is still necessary due to the following factors: 1) Determining a suitable window size prior to the application of any prediction method is essential [32]. Overly long window size may introduce some irrelevant features that would reduce predictive accuracy, and may take more computational time and memory space. 2) The HS^3^D dataset contains 2796/271,937 true/false donor sites (i.e., the ratio of true sites to false sites is almost 1:100). If all negative samples (false sites) are employed for building the prediction model, the huge number of training samples will increase the time complexity of some classifiers (e.g., SVM and ANN) [3, 33], and an extremely imbalanced class distribution will lead to poor predictive accuracy for some methods, for example, weighted matrix model (WMM) [9] and maximal dependency decomposition (MDD) [34]. If only a part of negative samples (e.g., 2796 negative samples [20]) are employed, predictive accuracy may be lost due to the underutilization of negative samples. 3) There are three billion DNA base pairs in the human genome, so the expected number of GT/AG is over 187 million. This abundance means that even a subtle improvement of the total predictive accuracy would drastically increase the absolute quantity of detected real splice sites.

In this study, we developed a computational approach to predict donor splice sites based on short window size and extremely imbalanced large samples. Our method, named chi-square decision table (χ^2^-DT), extracts the improved positional features based on chi-square tests, combines them with the frequencies of dinucleotides, and then designs a balanced decision table to predict the test samples, which can effectively resolve the imbalanced pattern classification problem. The results show that χ^2^-DT can achieve high predictive accuracy, high computational speed, and relatively good robustness against DNA sequencing errors (nucleotide insertions and deletions).

## Datasets and methods

### Datasets

We collected 2796/271,928 true/false donor splice sites from the publicly available HS^3^D dataset [35] (http://www.sci.unisannio.it/docenti/rampone/) for the experiments, and named them HS^3^D_all_. Each true/false donor splice site-containing sequence has 140 nucleotides, with the conserved dinucleotide GT at the 71st and 72nd positions, and does not contain non-ACGT bases. Setting the positions of the conserved GT as 00, the upstream positions were successively labeled as − 1, − 2, …, − 70, whereas the downstream positions were successively labeled as 1, 2, …, 68. From HS^3^D_all_, we randomly selected 796 true sites and 796 false sites to constitute a balanced testing set, named HS^3^D-test_1:1_, and then used the remaining sites to construct the training sets with different ratios of true sites to false sites. Additionally, to compare the performance of χ^2^-DT with that of other methods, we selected 2796 true sites and different numbers of false sites from HS^3^D_all_ to construct four datasets, namely, HS^3^D_I_, HS^3^D_II_, HS^3^D_III_, and HS^3^D_IV_.

The BG-570 dataset [36] (http://genome.crg.es/datasets/genomics96/) contains 570 human genomic DNA sequences and 570 corresponding mutated sequences. The mutated sequences were generated by introducing 1% random frameshift errors (nucleotide insertions and deletions) into the original DNA sequences. Using the BG-570 dataset, we constructed two testing sets (BG-570_orig_ and BG-570_muta_) to evaluate the robustness of χ^2^-DT against the frameshift errors. The extracting process of true/false sites in these two testing sets is described in the “Results and Discussion” section.

The numbers of true/false sites in the datasets described above are given in Table 1.

**Table 1:** Descriptions of various datasets

Datasets	Number of true donor sites	Number of false donor sites
HS^3^D_all_	2796	271928
HS^3^D_I_	2796	2796
HS^3^D_II_	2769	5000
HS^3^D_III_	2796	10000
HS^3^D_IV_	2796	15000
HS^3^D-test_1:1_	796	796
HS^3^D-train_1:1_	2000	2000
HS^3^D-train_1:10_	2000	20000
HS^3^D-train_1:20_	2000	40000
HS^3^D-train_1:50_	2000	100000
HS^3^D-train_1:135_	2000	271132
BG-570_orig_	2127	149039
BG-570_muta_	2081	149572

### Compressing 2 × 4 contingency table of each position with chi-square test

Just like Pearson correlation coefficient [37] and mutual information estimators [38] that are used for identifying relationships between variables, maximal information coefficient (MIC) [39] is a novel measure proposed to capture dependences between paired variables. For a pair of data series *x* and *y*, to calculate their MICvalue, ApproxMaxMI algorithm [39] sets *n*_*x*_ × *n*_*y*_ < *n*^0.6^ as the maximal grid size restriction; here, *n* is the sample size, and *n*_*x*_ and *n*_*y*_ are partition bins on *x* and *y*, respectively. Given *n* = 100, the MIC score for independent paired variables should be zero, and the corresponding partition should be a 2 × 2 grid. However, the ApproxMaxMI algorithm tends to fall into the maximal grid size (100^0.6^ ≈ 16), the corresponding partition is a 2 × 8 grid and the corresponding MIC score is 0.24, which leads to a nontrivial MIC score for independent paired variables under finite samples [40]. Recently, Chen et al. [40] presented the ChiMIC algorithm, which can control the excessive grid partitions of the ApproxMaxMI algorithm. Removing the maximal grid size limitation in ApproxMaxMI, ChiMIC uses a chi-square test based on a local *r* × 2 grid to determine whether the new endpoint should be introduced. If the *p*-value of the chi-square test is lower than a given threshold, the new endpoint is introduced for partition and ChiMIC continues searching for the next optimal endpoint. If the *p*-value of the chi-square test is greater than the given threshold, the new endpoint is discarded and the process of partition is terminated. For paired independent variables with *n* = 100, the MIC score calculated by ChiMIC is only about 0.06, and the corresponding partition is a 2 × 2 or 2 × 3 grid, clearly, the grid partition produced by ChiMIC is more reasonable.

**Fig. 1:**
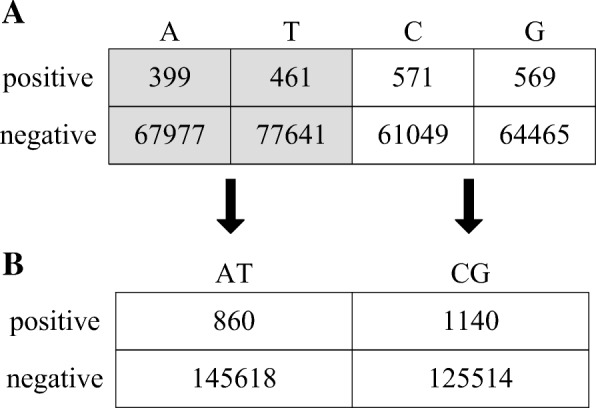
Compressing the 2 × 4 contingency table of position 6. **a**: 2×4 contingency table of position 6. **b**: 2×2 contingency table of position 6 after compression

Similarly, for each position in donor splice site-containing sequences, we can build a 2 × 4 contingency table to respectively count the frequencies of four bases in positive and negative samples. Figure 1a is the 2 × 4 table or 2 × 4 grid of position 6 based on HS^3^D-train_1:135_. Is the 2 × 4 table reasonable? Could it be compressed into a 2 × 3 table, or even a 2 × 2 table? For the local 2 × 2 contingency table (the light gray area in Fig. 1a), the *p*-value of the chi-square test is 0.8933 (> 0.01). This indicates that the endpoint between A and T should not be introduced according to the ChiMIC algorithm. In other words, that the base at position 6 is A or T cannot provide valuable information for distinguishing positive and negative samples. Similarly, the endpoint between C and G should not be introduced. Finally, the 2 × 4 contingency table of position 6 is compressed into a 2 × 2 contingency table (see Fig. 1b).

**Fig. 2:**
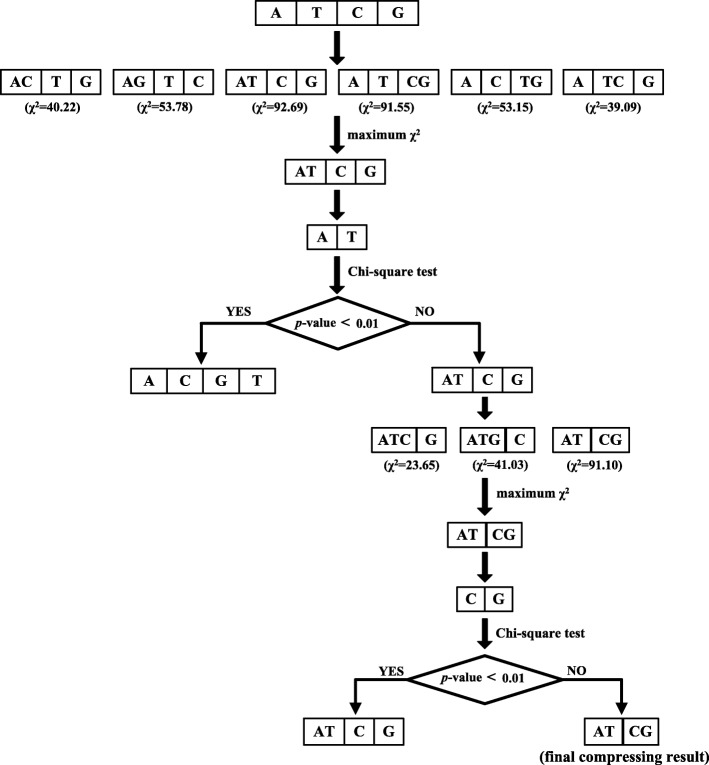
Illustration of compression procedure (position 6 in HS^3^D-train_1:135_)

The process of compressing the 2 × 4 contingency table of each position is described below. First, compress the 2 × 4 contingency table into six 2 × 3 contingency tables by merging any two different bases, and pick out the 2 × 3 contingency table that has the maximum chi-square value, denoted as *max*_2 × 3_. Next, reconstruct a local 2 × 2 contingency table based on the merged bases in *max*_2 × 3_ and perform a chi-square test. If the *p*-value is lower than a given threshold, *max*_2 × 3_ is unreasonable and should be backtracked to the 2 × 4 contingency table; then, the compression process is terminated. If the *p*-value is greater than a given threshold, *max*_2 × 3_ is reasonable; then, try to compress *max*_2 × 3_ into a 2 × 2 contingency table following the two steps above. Figure 2 further illustrates the compression procedure in detail.

### Window size determination

**Fig. 3:**
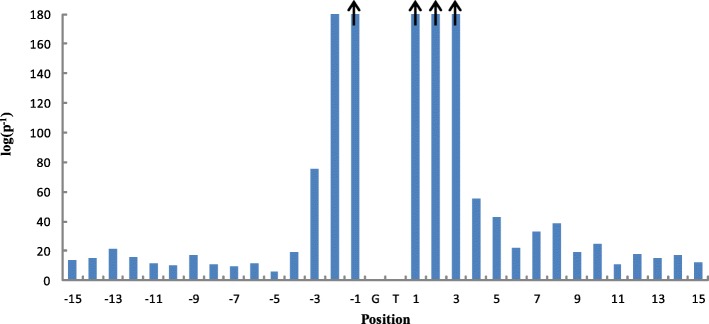
*log*(*p*^− 1^) values for different position**s.** ↑:The columns with arrows represent that *log*(*p*^− 1^) values of the corresponding positions are higher than that of position − 2. For simplicity, we just present the *log*(*p*^− 1^) values of positions − 15 to + 15

For each position in the sequences of 140 bp, we obtain a 2 × *r* contingency Table (2 ≤ *r* ≤ 4) after compression based on HS^3^D-train_1:135_; then, we perform a chi-square test with the 2 × *r* contingency table and calculate the logarithm of the reciprocal of *p*-value, here denoted as *log*(*p*^− 1^) (see Fig. 3). Higher *log*(*p*^− 1^) values mean that the corresponding positions are more important for discriminating positives from negatives. Therefore, we determine 11 bp (positions − 3 to + 8, excluding GT at positions 00) as the window size for donor splice site prediction. In the following text, the study will be based on the window size of 11 bp unless otherwise specified.

### Feature extraction

From each sample (a sequence of 11 bp in length), we extract 11 positional features and 16 compositional features. The compositional features are the frequencies of dinucleotides, which range from 0 to 10 because the sequence sample is only 11 bp. For each feature, we can obtain a 2 × r contingency Table (2 ≤ r ≤ 11) after compression; then, the status values of each feature correspond to *r* columns of the 2 × *r* table. For example, based on HS^3^D-train_1:135_, position 6 whose original status values are {A, C, G, T} corresponds to a 2 × 2 contingency table after compression, so it has two status values as {AT, CG}; the frequency of dinucleotide AA whose original status values are {0, 1, …, 10} corresponds to a 2 × 3 contingency table after compression, so it has three status values as {0, 1, 2–10}. It should be noted that, for the compositional features, only the adjacent original status values can be merged together in compression because they are sequential.

### Feature introduction

Suppose the proportion of the *k*^*th*^ class samples in sample set *D* is *p*_k_ (*k* = 1,2); then, the information entropy of *D* is defined as:

1 $$ H(D)=-\sum \limits_{k=1}^2{p}_k{\log}_2{p}_k $$

Given a feature *X*_*i*_ (1 ≤ *i* ≤ 27) that has *r*(2 ≤ *r* ≤ 11) status values as {*s*_1_, *s*_2_, …, *s*_*j*_, …, *s*_*r*_}, the information gain [41] that *X*_*i*_ brings for *D* can be calculated by:

2 $$ Gain\left(D,{X}_i\right)=H(D)-\sum \limits_{j=1}^r\frac{\mid {D}^j\mid }{\mid D\mid }H\left({D}^j\right) $$

where *D*^*j*^ represents the samples in *D* whose *X*_*i*_ takes the status value as *s*_*j*_(1 ≤ *j* ≤ *r*), while *H*(*D*^*j*^) is the information entropy of *D*^*j*^.

From the features whose information gains are above the average level, we pick out the one that has the highest gain ratio to be the first introduced feature. Here, the gain ratio of *X*_*i*_ is defined as:

3 $$ GainRatio\left(D,{X}_i\right)=\frac{Gain\left(D,{X}_i\right)}{IV\left({X}_i\right)} $$

where

4 $$ IV\left({X}_i\right)=-\sum \limits_{j=1}^r\frac{\left|{D}^j\right|}{\left|D\right|}{\log}_2\frac{\left|{D}^j\right|}{\left|D\right|} $$

and *IV*(*X*_*i*_) is the intrinsic value of *X*_*i*_.

Next, we introduce the remaining features one by one as follows.

- Step 1: Under the conditions in which the introduced features have existed, further compress the 2 × *r* contingency table of each remaining feature, in accordance with the compression process previously described. If the *r* columns are compressed into one column, the remaining feature cannot be introduced. If the *r* columns are not compressed into one column, the remaining feature is a candidate feature to be introduced.
- Step 2: Calculate the information gain of every candidate feature. Then, from the candidate features whose information gains are above the average level, pick out the one with the highest gain ratio to be the next introduced feature.
- Step 3: Repeat steps 1 and 2 until no feature can be introduced.

### Decision table design

The introduced features with their status values will form various decision rules. Taking HS^3^D-train_1:135_ as an example, 27 introduced features (including 11 positional features and 16 compositional features) have formed 201 decision rules (see Additional file 1: Table S1). We separately count the numbers of positive and negative samples that conform to the decision rules, and then construct a 2 × 201 imbalanced decision table (Table 2). In Table 2, the decision rule “(*P*_3_ = A)∧(*P*_− 1_ = ACT)∧(0 ≤ *f*_*GT*_ ≤ 2)” represents position 3 taking a value of A and position − 1 taking a value of ACT, while the frequency of dinucleotide GT takes values from 0 to 2. Other decision rules have similar representations. Given that the number of negative samples far exceeds that of positive samples, to resolve the imbalanced pattern classification problem, we adjust the decision weight of negative samples in each column of Table 2, i.e., multiply the number of negative samples in each column by *θ* (here, *θ* = 2000/271,132), and then get a 2 × 201 balanced decision table (Table 3).

**Table 2:** Imbalanced decision table based on HS^3^D-train_1:135_

Sample	Decision rule			Total
	(*P*_3_ = A)∧(*P*_−1_ = ACT)∧(0 ≤ *f*_*GT*_ ≤ 2)	…	(*P*_3_ = G)∧(*P*_− 1_ = G)∧(*P*_1_ = G)∧(*P*_2_ = G)∧(*P*_4_ = T) ∧(*P*_−2_ = CGT)	
positive	5	…	11	2000
negative	47,512	…	368	271,132

**Table 3:** Balanced decision table based on HS^3^D-train_1:135_

Sample	Decision rule			Total
	(*P*_3_ = A)∧(*P*_−1_ = ACT)∧(0 ≤ *f*_*GT*_ ≤ 2)	…	(*P*_3_ = G)∧(*P*_−1_ = G)∧(*P*_1_ = G)∧(*P*_2_ = G)∧(*P*_4_ = T) ∧(*P*_−2_ = CGT)	
positive	5	…	11	2000
negative (adjusted)	350.5	…	2.7	2000

When using the balanced decision table for making decisions, suppose a test sample meets the decision rule “(*P*_3_ = A)∧(*P*_− 1_ = ACT)∧(0 ≤ *f*_*GT*_ ≤ 2)”, first we assume that it is positive, replace 5 with 5 + 1, and calculate the corresponding chi-square value ${\upchi}_{i+}^2$. Then, we assume that it is negative, replace 350.5 with 350.5 + 1, and calculate the corresponding chi-square value ${\upchi}_{i-}^2$. If ${\upchi}_{i+}^2>{\upchi}_{i-}^2$, the test sample is predicted to be positive; otherwise, it is predicted to be negative. The decision process based on an imbalanced decision table is similar.

### Performance evaluation

Sensitivity (SN), specificity (SP), and the Matthew correlation coefficient (MCC) as common measures for evaluating binary classifications are defined as follows:

5 $$ SN=\frac{TP}{TP+ FN} $$

6 $$ SP=\frac{TN}{TN+ FP} $$

7 $$ MCC=\frac{TP\times TN- FN\times FP}{\sqrt{\left( TP+ FN\right)\times \left( TP+ FP\right)\times \left( TN+ FP\right)\times \left( TN+ FN\right)}} $$

Here, TP, FP, TN, and FN denote the numbers of true positives, false positives, true negatives, and false negatives, respectively. SN represents the percentage of positive samples correctly predicted as true. SP represents the percentage of negative samples correctly predicted as false. MCC takes into account true and false positives and negatives, and is generally regarded as a balanced measure. However, when the class distribution of the testing set is imbalanced, the MCC value will become relatively small, which cannot really reflect the performance of a classification model.

The global accuracy index Q^9^ [42] is independent of the class distribution and has been used by some researchers to evaluate the classifier performance in splice site prediction. Therefore, in this study, we choose Q^9^ as the measure of global accuracy to assess predictive performance in the case of an imbalanced testing set. Q^9^ is defined as follows:

8 $$ {Q}^9=\left(1+{q}^9\right)/2 $$

where

$$ {q}^9=\left\{\begin{array}{l}\left( TN- FP\right)/\left( TN+ FP\right),\\ {}\left( TP- FN\right)/\left( TP+ FN\right),\\ {}1-\sqrt{2}\sqrt{{\left[ FN/\left( TP+ FN\right)\right]}^2+{\left[ FP/\left( TN+ FP\right)\right]}^2,}\end{array}\begin{array}{l} if\  TP+ FN=0\\ {} if\  TN+ FP=0\\ {} if\ \mathrm{TP}+\mathrm{FN}\ne 0\ \mathrm{and}\ \mathrm{TN}+\mathrm{FP}\ne 0\end{array}\right. $$

The receiver operating characteristic (ROC) curve, which is widely used in evaluating the predictive accuracy of statistical predictors, is given by SN against 1 − SP. When dealing with highly skewed datasets, the Precision–Recall (PR) curve can provide better insight into an algorithm’s performance [43]. The areas under ROC and PR curves are denoted by AUC-ROC and AUC-PR, respectively. AUC-ROC and AUC-PR are estimated using the Davis–Goadrich method [43]. The closer the values of AUC-ROC and AUC-PR get to 1, the better the prediction model.

## Results and discussion

### Advantage with the short window size of 11 bp

Based on HS^3^D-train_1:1_ and HS^3^D-test_1:1_, the independent tests were performed to compare the performance of χ^2^-DT using various window sizes. The results (Table 4) show the following: 1) Comparing with the longer window sizes (e.g., 20 bp, 40 bp, 138 bp), χ^2^-DT with the window size of 11 bp can achieve the higher independent test accuracy. This indicates that overly long window sizes may introduce some irrelevant sequence information, thereby reduce prediction accuracy. 2) Short window size reduces feature dimension and saves computational time. For example, using the window size of 138 bp, the feature dimension is 154 (138 positional features and 16 compositional features); however, using the window size of 11 bp, the feature dimension drops to 27 (11 positional features and 16 compositional features), and there is about a 96% decrease in the elapsed time, running in the same computer system (Intel Core i5-3320M 2.6 GHz/8 GB RAM). Therefore, we have more confident on the short window size of 11 bp. The follow-up results are all based on 11-bp-long window size.

**Table 4:** Independent test accuracy based on various window sizes

Window size	Feature dimension	SN (%)	SP (%)	(SN + SP)/2(%)	MCC	Time (mm:ss)
11 bp(−3~ + 8)	27	93.09	91.58	92.34	0.847	00:18
20 bp(−10~ + 10)	36	93.34	90.95	92.15	0.843	00:24
40 bp(−20~ + 20)	56	91.33	91.83	91.58	0.832	01:09
138 bp(−70~ + 68)	154	92.71	89.45	91.08	0.822	07:18

### Superior performance with large extremely imbalanced dataset

For HS^3^D-test_1:1_, we respectively used imbalanced and balanced decision tables that were built based on various HS^3^D training sets to make decisions. The independent test results are given in Table 5.

**Table 5:** Independent test accuracy based on imbalanced and balanced decision tables

Training set	SN (%)		SP (%)		(SN + SP)/2 (%)		MCC	
	*imbal*	*bal*	*imbal*	*bal*	*imbal*	*bal*	*imbal*	*bal*
HS^3^D-train_1:1_	93.09	93.09	91.58	91.58	92.34	92.34	0.847	0.847
HS^3^D-train_1:10_	81.53	94.35	96.36	91.08	89.51	92.71	0.788	0.855
HS^3^D-train_1:20_	78.14	93.59	96.98	92.46	87.56	93.03	0.765	0.861
HS^3^D-train_1:50_	76.76	94.22	96.98	92.34	86.87	93.28	0.753	0.866
HS^3^D-train_1:135_	68.84	93.97	97.61	92.71	83.23	93.34	0.694	0.867

*imbal*. Denotes imbalanced decision table and *bal*. denotes balanced decision table

The results indicate the following: 1) When training sets are imbalanced, a balanced decision table can accurately predict donor splice sites. For a balanced decision table, MCC remains stable (0.847–0.867) with training sets having different positive-to-negative ratios. By contrast, for an imbalanced decision table, MCC continually drops with an increase in negative training samples, and declines from 0.847 (HS^3^D-train_1:1_) to 0.694 (HS^3^D-train_1:135_). Therefore, the follow-up results are produced by using balanced decision Tables. 2) Taking full advantage of training samples can improve predictive accuracy. Using a balanced decision table, MCC keeps on growing as the number of negative samples increases, and when the negative sample quantity peaks (271,132), MCC is at its highest (0.867).

Based on the same input features (11 positional features and 16 compositional features), χ^2^-DT was compared with the traditional classifiers including RF, ANN, and relaxed variable kernel density estimator (RVKDE) [44]. We selected RVKDE as a classifier for comparison because it can deliver the same level of accuracy as SVM and has lower time complexity when the training set is too large. We used Weka 3.8.1 software (https://www.cs.waikato.ac.nz/ml/weka/index.html) and the neural network toolbox [45] of Matlab R2015a to build RF and ANN classifiers, respectively, and all of the parameters took default values. The performance comparisons still employed the independent tests based on the HS^3^D-test_1:1_, HS^3^D-train_1:1_, and HS^3^D-train_1:135_; the corresponding results are given in Table 6.

**Table 6:** Independent test accuracy based on different classifiers

Classifier	SN (%)		SP (%)	(SN + SP)/2 (%)		MCC		
	HS^3^D-train_1:1_	HS^3^D-train_1:135_	HS^3^D-train_1:1_	HS^3^D-train_1:135_	HS^3^D-train_1:1_	HS^3^D-train_1:135_	HS^3^D-train_1:1_	HS^3^D-train_1:135_
RF	94.77	16.58	91.31	99.87	93.04	58.23	0.862	0.297
ANN	91.58	12.06	91.83	99.91	91.71	55.98	0.834	0.248
RVKDE	96.23	23.37	88.82	99.50	92.53	61.43	0.853	0.353
χ^2^-DT	93.09	93.97	91.58	92.71	92.34	93.34	0.847	0.867

The results indicate the following: 1) Using the extremely imbalanced training set, χ^2^-DT outperforms all of the other classifiers. As Table 6 shows, based on HS^3^D-train_1:1_, MCC of χ^2^-DT is 0.847, which is comparable to those of RF, ANN, and RVKDE. In contrast, based on HS^3^D-train_1:135_, MCC of χ^2^-DT rises to 0.867, and is significantly higher than those of the other classifiers (0.248–0.353). 2) With the large training set, χ^2^-DT has an advantage with regard to computational speed. We ran all of the simulations on an Intel Core i5-3320M 2.6 GHz/8 GB RAM system. For HS^3^D-train_1:135_, the elapsed time of χ^2^-DT was just 89 s, while RVKDE took more than 32 h. This speed of χ^2^-DT is due to the fact that no parameters need to be optimized.

### Good robustness against DNA sequencing errors

In BG-570 dataset, setting the window size as 11 bp (including positions − 3 to − 1 upstream of the conserved GT and positions + 1 to + 8 downstream of it, but excluding the conserved GT), we can extract 2127/149,039 true/false donor splice site-containing sequences from 570 original DNA sequences to constitute a testing set called BG-570_orig_, and extract 2081/149,572 true/false donor splice site-containing sequences from 570 mutated DNA sequences to constitute another testing set called BG-570_muta_. Based on HS^3^D-train_1:135_, the independent test results respectively employing the positional features and the combination of positional and compositional features are shown in Table 7.

**Table 7:** Independent test accuracy based on different features

Testing set	Feature	SN (%)	SP (%)	(SN+SP)/2 (%)	MCC	Q^9^ (%)
BG-570_orig_	positional	93.09	92.11	92.60	0.349	92.58
	positional+compositional	93.51	92.15	92.83	0.352	92.70
BG-570_muta_	positional	90.55	91.77	91.16	0.329	91.14
	positional+compositional	92.67	92.12	92.40	0.344	92.39

The MCC values in Table 7 are low (0.329–0.352) due to the highly imbalanced testing sets. To effectively assess predictive performance, the global accuracy index Q^9^, which is invariant to class skew, is added for evaluation purposes. The comparative results demonstrate that: 1) The compositional features have tolerance to frameshift errors of DNA sequencing. Based on the positional features, Q^9^obtained under the testing of BG-570_muta_ is 0.9114, lower than that under BG-570_orig_ (0.9258). However, after adding compositional features, Q^9^ rises back to 0.9239 when still tested by BG-570_muta_. 2) Whether or not there are frameshift errors in testing sets, χ^2^-DT can achieve satisfactory performance (Q^9^ ≥ 0.92).

### Better performance in comparison with existing methods

10-fold cross validation was applied to assess the predictive performance of χ^2^-DT, with the aim of comparing it with existing methods. To perform 10-fold cross validation, the dataset was randomly divided into ten non-overlapping subsets of equal size. In each repetition, one subset was used as a testing set and the remaining nine subsets were used as a training set. Based on each training set, we built a balanced decision table independently. The average of ten values of predictive accuracy was used as the final accuracy. All comparisons were carried out in the HS^3^D datasets, and the 10-fold cross accuracy values of the methods for comparison were obtained directly from the corresponding references.

On the one hand, χ^2^-DT was compared with the methods using longer window size (≥100 bp), including a first-order Markov model combined with a dinucleotide-based hidden Markov model (MM1-H2MM) [31], SVM with a Bayes kernel (SVM-B) [25], and Meher’s method [13]. The web server (MaLDoSS) based on Meher’s method is available at http://cabgrid.res.in:8080/maldoss. The 10-fold cross accuracy of χ^2^-DT was calculated based on HS^3^D_all_. Table 8 shows that χ^2^-DT with much shorter window size can achieve better predictive performance, despite the degree of imbalance of the training set being higher.

**Table 8:** 10-fold cross accuracy based on comparisons with the long-window size-based methods

Method	Window size (bp)	Ratio of positive-to-negative samples	SN (%)	SP (%)	(SN + SP)/2 (%)	Q^9^ (%)
MM1-H2MM	140	2796:27960 (1:10)	93.81	91.69	92.75	92.63
SVM-B	140	2796:27960 (1:10)	94.13	90.99	92.56	92.39
Meher’s method	102	2796:53124 (1:19)	88.30	89.40	88.90	88.80
χ^2^-DT	11	2796:271928 (1:97)	94.11	92.58	93.35	93.30

On the other hand, χ^2^-DT was compared with the methods using short window size (9 bp). Maximum entropy model (MEM) [46] and SAE [12] are the typical methods for predicting donor splice sites using short window size. The web server (MaxEntScan) based on MEM is available at http://genes.mit.edu/burgelab/maxent/Xmaxentscan_scoreseq.html. The web server based on SAE is available at http://cabgrid.res.in:8080/sspred. Based on the HS^3^D datasets with different ratios of positive-to-negative samples (i.e., 2796:2796, 2796:5000, 2796:10000, 2796:15000), the AUC-ROC and AUC-PR values of SAE, MEM, WMM, MDD and first-order Markov model (MM1) were calculated by MaxEntScan, employing 9-bp-long window size. For comparison, we also calculated the AUC-ROC and AUC-PR values of χ^2^-DT based on HS^3^D_I_, HS^3^D_II_, HS^3^D_III_, and HS^3^D_IV_. The results (Table 9) showed that the predictive performance of χ^2^-DT was clearly superior to those of all of the other methods. As the degree of imbalance of the dataset increased, the AUC-PRs of all of the methods continuously declined, partly due to the fact that the evaluation indicator AUC-PR is sensitive to class skew. However, for the other methods besides χ^2^-DT, their AUC-PRs declined more dramatically. For example, when the degree of imbalance peaked (2796:15,000), AUC-PRs of the other methods were around 0.68, with decline of up to 28%, while the AUC-PR value of χ^2^-DT was 0.85, representing a decline of only about 10%.

**Table 9:** 10-fold cross accuracy based on comparisons with the short-window size-based methods

Method	AUC-ROC(±SE)				AUC-PR(±SE)			
	2796:2796	2796:5000	2796:10000	2796:15000	2796:2796	2796:5000	2796:10000	2796:15000
SAE	0.946 (±0.0031)	0.945 (±0.0031)	0.944 (±0.0030)	0.945 (±0.0030)	0.945 (±0.0031)	0.876 (±0.0045)	0.772 (±0.0055)	0.682 (±0.0059)
MEM	0.948 (±0.0031)	0.946 (±0.0031)	0.947 (±0.0030)	0.947 (±0.0030)	0.947 (±0.0031)	0.878 (±0.0045)	0.773 (±0.0055)	0.683 (±0.0059)
MDD	0.945 (±0.0031)	0.942 (±0.0032)	0.944 (±0.0030)	0.944 (±0.0030)	0.944 (±0.0031)	0.872 (±0.0046)	0.769 (±0.0055)	0.680 (±0.0059)
MM1	0.945 (±0.0031)	0.941 (±0.0032)	0.936 (±0.0032)	0.941 (±0.0031)	0.942 (±0.0032)	0.870 (±0.0046)	0.765 (±0.0056)	0.679 (±0.0060)
WMM	0.927 (±0.0036)	0.924 (±0.0036)	0.924 (±0.0035)	0.925 (±0.0034)	0.924 (±0.0037)	0.867 (±0.0046)	0.703 (±0.0060)	0.675 (±0.0060)
χ^2^-DT	0.965 (±0.0023)	0.969 (±0.0027)	0.971 (±0.0025)	0.971 (±0.0025)	0.953 (±0.0030)	0.932 (±0.0034)	0.879 (±0.0042)	0.856 (±0.0038)

*SE* Standard error

## Conclusions

Based on the short window size of 11 bp, a high-performance method for predicting donor splice sites, called χ^2^-DT, was proposed. In terms of accuracy, χ^2^-DT is clearly superior to the methods for comparison. With regard to computational speed, χ^2^-DT is fast, even when using a large training set with more than 270,000 samples, because no parameters need to be optimized during model training. Furthermore, the independent test results based on the BG-570 dataset indicate that χ^2^-DT has relatively good robustness against frameshift errors in DNA sequencing, due to the addition of compositional features.

In future research, we plan to focus on the following: 1) We will attempt to combine more valuable features (e.g., DNA structural properties) for characterizing the candidate splice sites, in pursuit of better predictive performance. 2) When χ^2^-DT is applied to predicting acceptor splice sites, it does not further improve the predictive accuracy of existing methods, so it is necessary to devise another optimal model for acceptor sites. 3) The detection of splice sites ultimately involves identifying genes, so our overall goal is to constantly improve the proposed splice site predictor, and then use it to find genes.

## Reviewers’ comments

### Reviewer’s report 1

Ryan McGinty, Ph.D.

## Reviewers’ comments

Reviewer summary:

Zeng, et al. have created a new computational method, X2-DT, for predicting gene splice donor sites that uses a very small window size (11 bp), is robust to a very low true/false ratio in their training data set, and runs efficiently. This method appears to perform as well or better than previous methods which were compared in this study. It would benefit the manuscript for the authors to make a clearer case for the usefulness and applicability of their tool.

Reviewer recommendations to authors:

Major suggestions: Clarify how X2-DT could be used by others and why it would be useful. The authors state that X2-DT can be used for the “prediction of splice sites in short reads generated by next-generation sequencing.” However, it is never stated whether it should be applied to short reads generated from genomic sequencing or transcriptome sequencing. From the context, it would appear to be the latter, as genomic short read sequences are assembled into longer fragments and therefore the read length is irrelevant to the window size. Incidentally, there also exists a field dedicated to predicting splice site strength from genomic sequences, rather than RNA sequences. Assuming the short reads in question here are from transcriptome sequencing, the issue that the authors propose to solve can be described more clearly. In this case, the short reads should contain spliced mRNA sequences, and so the issue becomes whether there is enough sequence context on either side of the splice site to unambiguously map the splice junction without the need for computational prediction. The authors suggest that “high-throughput DNA sequencing technologies produce billions of short reads with lengths of about 50 bp [32], while most splice site prediction methods need long sequences (≥100 bp).”In fact, the read length of the most commonly-used platform varies from 50 to 150 bp or more, and paired-ends can be utilized to increase the likelihood of capturing a splice junction. A 2015 study [“The impact of read length on quantification of differentially expressed genes and splice junction detection.” Chhangawala, et al.] finds that “there is little difference for the detection of differential expression regardless of the read length,” however, “splice junction detection significantly improves as the read length increases.” From this, we can assume that someone designing a sequencing study to discover splice junctions and other features of the transcriptome would have generated > 100 bp paired-end reads from the outset, and would not benefit greatly from the new tool presented here. However, the authors could highlight the usefulness of X2-DT in discovering splice junctions from sequencing studies where differential expression rather than transcriptome profiling was the initial aim of the study, and thus shorter reads were generated. To this end, I would suggest the authors conduct the following analyses: First, perform a parallel analysis of the same data used in Chhangawala, et al. 2015 (see above), showing the ability of X2-DT to augment splice site detection from RNA-seq data of various read lengths. For instance, does running X2-DT on 50 bp reads find as many splice sites as 100 bp reads without X2-DT? Does X2-DT improve on 100 bp reads at all? The authors could thus make the case for using their tool in very practical terms by showing that it is the equivalent of adding N bp to the sequencing read length. Next, perform a meta-analysis of the read lengths used across all RNA-seq studies, to show the magnitude of the untapped source of new splice junctions in existing RNA-seq data, which can now be found due to the unique short-window analysis of X2-DT. Combined with the above new analysis, it may be possible to estimate how many novel splice junctions can be found per transcriptome, how many transcriptomes currently exist to be analyzed, and therefore some rough estimate of the potential biological impact of this study.

Authors’ response: *We appreciate the detailed recommendations made by the reviewer. This study is limited to DNA sequence data generated from genomic sequencing. As mentioned by the reviewer, genomic short read sequences are assembled into longer fragments before splice site prediction, so it is inappropriate to highlight the argument that χ*^*2*^*-DT can predict the splice sites in short reads generated by next-generation sequencing. In the revised manuscript, we removed this inappropriate argument.*

However, for our method, the use of short window size (11 bp) is necessary as far as improving prediction accuracy and simplifying the prediction model. In the revised manuscript, we discussed the benefits that 11-bp-long window size brought. Based on HS^3^D-train_1:1_ and HS^3^D-test_1:1_, the independent tests were performed to compare the performance of χ^2^-DT using various window sizes. The results (Table 10) show the following: 1) Comparing with the longer window sizes (e.g., 20 bp, 40 bp, 138 bp), χ^2^-DT with 11-bp-long window size can achieve higher independent test accuracy. This indicates that overly long window sizes may introduce some irrelevant sequence information, thereby reduce the prediction accuracy. 2) Short window size reduces feature dimension and saves computational time. For example, based on 138-bp-long window size, the feature dimension is 154 (138 positional features and 16 compositional features); while, as for 11 bp-long window size, the feature dimension drops to 27 (11 positional features and 16 compositional features), and there is about a 96% decrease in the elapsed time, running in the same computer system.

**Table 10:** Independent test accuracy based on various window sizes

Window size	Feature dimension	SN (%)	SP (%)	(SN + SP)/2(%)	MCC	Time (mm:ss)
11 bp(−3~ + 8)	27	93.09	91.58	92.34	0.847	00:18
20 bp(−10~ + 10)	36	93.34	90.95	92.15	0.843	00:24
40 bp(−20~ + 20)	56	91.33	91.83	91.58	0.832	01:09
138 bp(−70~ + 68)	154	92.71	89.45	91.08	0.822	07:18

Additionally, as described in the results and discussion part of manuscript, χ^2^-DT using 11-bp-long window size was compared with several existing approaches that used longer window sizes (e.g., 140 bp). The results (Table 8) indicate that χ^2^-DT obtains better predictive performance.

From the results above, we have more confident on the short window size we used. Though, we still believe that correct identification depends more on the proposed method itself. As shown in the results and discussion part of manuscript, when compared with three traditional classifiers (RF, ANN, RVKDE) that are inputted the same feature vectors, our method obtains higher prediction accuracy (see Table 6); when compared with other splice site prediction approaches that also used short window size (e.g., 9 bp), our method is found to perform better (see Table 9). Therefore, although many computational methods have been developed presently for predicting splice sites, our method provides a supplement to the commonly used splice site prediction methods because of its high performance, and is believed to contribute to the prediction of eukaryotic gene structure.

Necessary changes have been made in the revised manuscript: Title of manuscript, Abstract, Keywords, page 4 (lines 108–110), page 5 (line 123), page 8 (lines 200–201), page 12 (lines 294–307), page 15 (lines 373,378,381–383), page 16 (line 401), titles of Tables 8 and 9; we add Table 4 and reference [32].

Minor issues:

The authors compare their work to several existing methods. While these methods are categorized and listed by their method or strategy, it would be of some practical use to know the name of each tool being compared in each table. Stylistically, I would prefer more detailed explanations of the methods that might help the study be understood by a broader audience. As written, there is a heavy prerequisite for knowledge of statistical and computational methods, including much undefined terminology. This knowledge is likely not shared by many readers interested in the biology of splicing, or with a practical need to employ the best splicing prediction program.

Authors’ response: *SVM-B, WMM, MDD, MM1 and MEM are the conventional models for predicting splice sites, and they are often employed for comparing with new presented methods. In Table** 9**, the results of MEM, MDD, WMM and MM1 were obtained by executing the MaxEntScan (a web server) which is available at* *http://genes.mit.edu/burgelab/maxent/Xmaxentscan_scoreseq.html**. Meher’s method and SAE are recently developed methods for donor splice site prediction. Based on Meher’s method, a web server (MaLDoSS) has been developed, and is available at* *http://cabgrid.res.in:8080/maldoss**. The web server based on SAE can be available at* *http://cabgrid.res.in:8080/sspred**. As per suggestion, we supplement the above contents in the revised manuscript for the convenience of practical application. As for written, we have checked and revised carefully, to avoid undefined terminology, in the hope that many readers interested in this study can understand.*

Necessary changes have been made in the revised manuscript: page 15 (lines 364, 375–377, 383), page 16 (lines 384–388).

### Reviewer’s report 2

Dirk Walther

## Reviewers’ comments

Reviewer summary:

Prediction of splice-sites has been a long-standing problem in Bioinformatics and many algorithms have been developed, essentially exhausting all possible ways to formulate and solve the computational problem. Despite the many methods and their reasonable success, and despite the increased availability of transcript sequencing data which allow determining splices sites based on experimental information, this reviewer is willing to be open to new in-silico methods. Clearly, correct splice site prediction would help tremendously for genome annotation purposes.

Reviewer recommendations to authors:

(1) The authors highlight as an advantage and pose as a need to base predictions on short sequence motifs (11mers) as necessitated by the short available sequence reads from DNAseq data. Though, I would think, splices site predictions would always be applied to assembled genomes or genes, not individual reads. So for me, this is not an argument at all. The length of the k-mer should reflect what is truly necessary for correct identifications. That aside, I still believe it is interesting to see how well methods based on short k-mers can work.

Authors’ response: *We agree with the recommendations given by the reviewer. In the revised manuscript, we removed the inappropriate argument that χ*^*2*^*-DT can predict the splice sites in short reads generated by next-generation sequencing. And we changed “short sequence” in the title to “short window size” which we thought may be more appropriate.*

In this study, the use of short window size (11 bp) is necessary as far as improving prediction accuracy and simplifying the prediction model. In the revised manuscript, we discussed the benefits that 11-bp-long window size brought. Based on HS^3^D-train_1:1_ and HS^3^D-test_1:1_, the independent tests were performed to compare the performance of χ^2^-DT using various window sizes. The results (Table 11) show the following: 1) Comparing with the longer window sizes (e.g., 20 bp, 40 bp, 138 bp), χ^2^-DT with 11-bp-long window size can achieve the highest independent test accuracy. This indicates that overly long window sizes may introduce some irrelevant sequence information, thereby reduce the prediction accuracy. 2) Short window size reduces feature dimension and saves computational time. For example, based on 138-bp-long window size, the feature dimension is 154 (138 positional features and 16 compositional features); while, as for 11-bp-long window size, the feature dimension drops to 27 (11 positional features and 16 compositional features), and there is about a 96% decrease in the elapsed time, when running in the same computer system.

**Table 11:** Independent test accuracy based on various window sizes

Window size	Feature dimension	SN (%)	SP (%)	(SN + SP)/2(%)	MCC	Time (mm:ss)
11 bp(− 3~ + 8)	27	93.09	91.58	92.34	0.847	00:18
20 bp(−10~ + 10)	36	93.34	90.95	92.15	0.843	00:24
40 bp(−20~ + 20)	56	91.33	91.83	91.58	0.832	01:09
138 bp(−70~ + 68)	154	92.71	89.45	91.08	0.822	07:18

Additionally, as described in the results and discussion part of manuscript, χ^2^-DT using 11-bp-long window size was compared with several existing approaches that used long window sizes (e.g., 140 bp). The results (Table 8) indicate that χ^2^-DT obtains better predictive performance.

Necessary changes have been made in the revised manuscript: Title of manuscript, Abstract, Keywords, page 4 (lines 108–110), page 5 (line 123), page 8 (lines 200–201), page 12 (lines 294–307), page 15 (lines 373,378,381–383), page 16 (line 401), titles of Tables 8 and 9; we add Table 4 and reference [32].

(2) The study reports results on donor sites only. The authors state that with regard to acceptor sites, no performance gain has been achieved, leading me to believe that performance was at least comparable. This should be discussed more - why gain for donor sites, not acceptor sites. Also, this point should be mentioned much sooner in the manuscript than in the very last paragraph. Furthermore, the equal performance of their method relative to others should be documented.

Authors’ response: *We determined the 18-bp-long window size (− 17~ + 1) by chi-square test, for predicting acceptor splice sites. Using 2880/28,800 true/false acceptor splice sites from HS*^*3*^*D dataset, 10-fold cross validation was applied to assess the performance of χ*^*2*^*-DT, and the predictive accuracy is: SN = 0.8901, SP = 0.8751, Q*^*9*^ *= 0.8826. Based on the same dataset, Q*^*9*^*achieved by SVM-B and MM1-H2MM are 0.8951 and 0.9057, respectively, which are slightly higher than that of our method.*

χ^2^-DT employs positional features and compositional features. While, as for acceptor sites, we found positional features and compositional features were not enough to characterize the candidate samples, maybe some other valuable features, such as DNA structural properties [19], should be involved. We are working on a new model for predicting acceptor splice sites with improved prediction accuracy, and the related researches will be reported in the forthcoming paper.

(3) The method section needs a better introduction/motivation. I had difficulties grasping the basic rationale of the method. In fact, I am not sure, I did. I could not follow the arguments with regard to “compressing that tables” at all. More explanation is needed.

Authors’ response: *Let’s begin with maximal information coefficient (MIC)* *[*39*]. Just like Pearson correlation coefficient* *[*37*]* *and mutual information estimators* *[*38*]* *that are used for identifying relationships between variables, MIC is a novel measure proposed to capture dependences between paired variables. Giving an independent paired variables {x*_*i*_*, y*_*i*_*}(i = 1,2,…20), x*_*i*_*, y*_*i*_*∈(0,1), as shown in following:*

To calculate the MIC value of *x* and *y*, a maximum grid solution (a 2 × 9 grid, i.e., *y* and *x* are respectively partitioned as 2 bins and 9 bins) with the highest induced mutual information will be searched. And a 2 × 9 table (Table 12) is generated for counting the number of the samples falling into each grid.

**Table 12:** 2 × 9 table for counting the number of the samples in each grid

	0 < *x* ≤ 0.05	0.05 < *x* ≤ 0.29	0.29 < *x* ≤ 0.55	0.55 < *x* ≤ 0.57	0.57 < *x* ≤ 0.62	0.62 < *x* ≤ 0.69	0.69 < *x* ≤ 0.71	0.71 < *x* ≤ 0.84	0.84 < *x* ≤ 0.85	0.85 < *x* < 1
0 < *y* ≤ 0.5	0	4	0	1	0	1	0	2	0	2
0.5 < *y* < 1	2	0	3	0	3	0	1	0	1	0

The MIC value of *x* and *y* calculated based on the 2 × 9 grid (2 × 9 table) will achieve 1, clearly, it is illogical, because MIC value should be tend to 0 for statistically independent variables. Thus, to avoid producing the nontrivial MIC values due to excessive grid partitions, ApproxMaxMI algorithm [39] sets *n*^0.6^ as the maximal grid size restriction, here, *n* is the sample size. Then, a 2 × 3 grid would be generated to partition data, and the corresponding MIC value falls to 0.31. So the 2 × 9 table is compressed into a 2 × 3 table (Table 13).

**Table 13:** 2 × 3 table for counting the number of the samples in each grid

	0 < x ≤ 0.05	0.05 < x ≤ 0.29	0.29 < x < 1
0 < *y* ≤ 0.5	0	4	6
0.5 < *y* < 1	2	0	8

Recently, our research group presented the ChiMIC algorithm [40] for calculating MIC value. ChiMIC uses a chi-square test based on a local *r* × 2 grid to determine whether the new endpoint should be introduced, and removes the maximal grid size limitation in ApproxMaxMI. For the example above, the grid partition generated by ChiMIC is a 2 × 2 grid, and the corresponding MIC value is only about 0.11 that is more close to 0. It means that further compressing the 2 × 3 grid (2 × 3 table) is reasonable.

Similarly, for each position in donor splice site-containing sequences, we can build a 2 × 4 contingency table to respectively count the frequencies of four bases in positive and negative samples. Is the 2 × 4 table reasonable? Could it be compressed into a 2 × 3 table, or even a 2 × 2 table? Taking position 6 as an example, its 2 × 4 contingency table is finally compressed into a 2 × 2 contingency table, according to the ChiMIC algorithm (see Fig. 1).

Moreover, if do not compress the 2 × 4 contingency table of each position, we will get a 2 × 4^11^ decision table after introducing 11 positional features, and with the further introduction of features, the number of columns in decision table will be grow exponentially, then the decision table would be quite sparse.

Therefore, we compressed the 2 × r contingency table of each feature, including positional and compositional features. And the results indicate the compression strategy is effective for correct prediction.

Necessary changes have been made in the revised manuscript: page 6 (lines 155–157), page 7 (lines 172–176); we add references [37, 38].

(4) Despite trying, I had difficulties understanding, where and how the imbalance was tested (during training or during testing or both?) Try to be more clear about it. So, in essence, I was not able to assess whether the claimed improved performance on this imbalanced problem was, in fact, achieved.

Authors’ response: *The number of false donor sites far exceeds that of true donor sites, e.g., the HS*^*3*^*D dataset contains 2796/271,937 true/false donor sites. If all negative samples (false sites) are employed for building the prediction model, the extremely imbalanced large training samples will lead to poor predictive results for many methods.*

We give an example to explain how we resolve the imbalanced pattern classification problem. Suppose there are 87/1687 positive/negative training samples, if only 2 positional features (position − 1 and 3) are introduced and have formed 4 decision rules, we separately count the numbers of positive and negative samples that conform to the decision rules, then get a 2 × 4 imbalanced decision table (Table 14).

**Table 14:** Imbalanced decision table

Sample	Decision rule				Total
	(*P*_−1_ = ACT)∧(*P*_3_ = AT)	(*P*_−1_ = ACT)∧(*P*_3_ = GC)	(*P*_− 1_ = G)∧(*P*_3_ = AT)	(*P*_− 1_ = G)∧(*P*_3_ = GC)	
positive	38	12	26	11	87
negative	184	1026	289	188	1687

Giving a positive testing sample, suppose its position −1 and 3 both take a value of G, the testing sample will conform to the decision rule “(*P*_− 1_ = G)∧(*P*_3_ = GC)”. In Table 14, replace 11 with 11 + 1, and calculate the corresponding chi-square value ${\upchi}_{i+}^2$ (109.2); similarly, replace 188 with 188 + 1, and calculate the corresponding chi-square value ${\upchi}_{i-}^2$ (110.1). Here, ${\upchi}_{i+}^2<{\upchi}_{i-}^2$, so this testing sample is wrongly predicted to be negative, according to the imbalanced decision table.

Now, we adjust the decision weight of negative samples in each column, i.e., multiply the number of negative samples in each column by 87/1687, and then get a balanced decision table (Table 15). In Table 15, replace 11 with 11 + 1, and calculate the corresponding chi-square value ${\upchi}_{i+}^2$ (46.2); replace 9.7 with 9.7 + 1, and calculate the corresponding chi-square value ${\upchi}_{i-}^2$ (45.9). Here, ${\upchi}_{i+}^2$ > ${\upchi}_{i-}^2$, so the testing sample is predicted to be positive. Therefore, in the case of imbalanced training set, the use of balanced decision table can correctly make decisions.

**Table 15:** Balanced decision table

Sample	Decision rule				Total
	(*P*_− 1_ = ACT)∧(*P*_3_ = AT)	(*P*_− 1_ = ACT)∧(*P*_3_ = GC)	(*P*_−1_ = G)∧(*P*_3_ = AT)	(*P*_−1_ = G)∧(*P*_3_ = GC)	
positive	38	12	26	11	87
negative	9.5	52.9	14.9	9.7	87

In this study, we use 2000/271,132 positive/negative samples to generate an extremely imbalanced training set (HS^3^D-train_1:135_), and use 796/796 positive/negative samples to generate a balanced testing set (HS^3^D-test_1:1_). The independent testing results (Table 6) based on HS^3^D-train_1:135_ and HS^3^D-test_1:1_ show that when training set is imbalanced, the MCC value of our method is 0.867, while the MCC value of other traditional classifiers (RF, ANN, and RVKDE) is about 0.25–0.35. Further, it is found in Table 5 that the MCC values obtained by balanced decision table keep on growing as the number of negative training samples increases, i.e., rise from 0.847 to 0.867, which indicates taking full advantage of training samples could improve predictive accuracy.

Necessary changes have been made in the revised manuscript: page 10 (lines 251–253).

Minor issues

Generally, the article is well written (English-wise). Some minor mistakes need correcting. For example, use present tense in the Abstract when talking about your method and results (“The proposed method presents” (not “presented”). Check use of the definite articles.

*Authors’ response: Following the suggestions of reviewer, we have made language corrections, including tense and use of the definite articles.*

Necessary changes have been made in the revised manuscript: Abstract, page 14 (line 346).

## Additional file

Additional file 1: **Table S1.** The table lists 201 decision rules obtained based on HS^3^D-train_1:135_, and lists the number of positive and negative training samples conforming to the decision rules. (XLSX 38 kb)

## Abbreviations

χ^2^-DT: Chi-square decision table
ANN: Artificial neural network
AUC-PR: Areas under PR
AUC-ROC: Areas under ROC
HS3D: *Homo Sapiens* Splice Sites Dataset
MCC: Matthew correlation coefficient
MDD: Maximal dependency decomposition
MEM: Maximum entropy model
MIC: Maximal information coefficient
MM1: first-order Markov model
MM1-H2MM: First-order Markov model combined with a dinucleotide-based hidden Markov model
PR: Precision–Recall
RF: Random forest
ROC: Receiver operating characteristic
RVKDE: Relaxed variable kernel density estimator
SAE: The sum of absolute error
SN: Sensitivity
SP: Specificity
SVM: Support vector machine
SVM-B: SVM with a Bayes kernel
WMM: weighted matrix model
